# A Brief Account of Splenectomy with a Case of Simple Hypertrophy of the Spleen Treated by Operation

**Published:** 1910-09

**Authors:** James Swain

**Affiliations:** Professor of Surgery in the University of Bristol; Surgeon to the Bristol Royal Infirmary.


### A BRIEF ACCOUNT OF SPLENECTOMY WITH A CASE OF SIMPLE HYPERTROPHY OF THE SPLEEN TREATED BY OPERATION.

BY

JAMES SWAIN, M.S., M.D. Lond., F.R.C.S.,

*Professor of Surgery in the University of Bristol;*
*Surgeon to the Bristol Royal Infirmary.*

AMONGST Greek and Roman athletes it was commonly supposed that the presence of the spleen was a hindrance to their power of running, and they used certain drugs which were thought to have the effect of diminishing or dissolving the organ. With the same object Hippocrates advised the application of a preparation of mushrooms.

In support of this supposed influence of the spleen some surgeons sprang into notoriety about 1700 from their operations of removing the milt or spleen—" unmilting," as it was then called—in order to improve the wind of the individual. Dionis, writing in 1733, speaks plainly on the foolishness of " these cruel operations, which, owing their existence to some crude brains, found a sepulture in that of their inventors." A survival of the practice exists to-day in the French proverb, " Courir comme un dérate."

Zaccarelli is said to have successfully removed the spleen for disease in 1549, and Matthias performed a similar operation for prolapse through a parietal wound from a stab in 1678; but the introduction of splenectomy into modern surgery may be said to date from 1867, when Péan successfully excised an enlarged and cystic spleen in a woman twenty years of age.

In 1895 Dr. Richard Douglas published a list of 194 splenectomies which he had collected. For leucocythæmic spleen there were thirty-six operations, with thirty-one deaths; for simple hypertrophy fifty-nine operations, with twenty-five deaths; for neoplasms, five operations with three deaths; for hydatids, six operations with two deaths; for wounds, forty-three operations with eleven deaths. In 1900 Hagen collected 360 cases of splenectomy, with 138 deaths, a mortality of 38 per cent. ; but the mortality before 1890 was 42 per cent., and dropped to 19 per cent. between 1890 and 1900. This diminution in mortality is largely owing to the recognition of the fact that splenectomy in leucocythemia is, as Bryant puts it, " physiologically unsound and surgically unsafe " ; for even in those few cases that have survived the operation there is no evidence that the patients have derived any benefit from its performance.

Speaking generally, the operation of splenectomy offers the best chance of permanent success when the disease is primarily splenic, and it should not be performed in general constitutional disorders of which the splenic enlargement is only a local and secondary manifestation.

For traumatic lesions, wandering spleen, simple and malarial hypertrophy, cysts, abscess, sarcoma, primary tuberculosis and Banti's disease, operation is justifiable ; but for myelogenous and lymphatic leukæmia, Hodgkin's disease, amyloid degeneration, syphilis, and the chronic enlargement of the spleen which occurs in infancy, the operation should be avoided.

Before deciding on operation an examination of the blood is necessary in most cases, in order that certain general diseases may be excluded. Splenectomy is the operation of choice, for it is generally safer to remove the whole organ than to adopt conservative operative measures.

Though many cases of splenectomy are on record, very few occur in the practice of any individual surgeon, and therefore the following case of simple hypertrophy is given as an example of one of the conditions for which operation is justifiable.

M.J., female, aged 26 years, was sent to me by Dr. Haslett, of Pontypool, in August, 1902. She had then recently had an attack of jaundice, and during that time the tumour was discovered in the left side of the abdomen. There was some pain over the swelling, and the patient complained of slight shortness of breath for the previous fortnight. Marked anorexia. She was rather spare, and apparently not anæmic. Occupying practically the whole of the left side of the abdomen was a smooth, movable, firm tumour, springing from the left hypochondrium, but capable of being pushed towards the pelvis, so that the top of the tumour could be felt. The anterior edge of the swelling could be taken between the fingers, and along this border, about two inches below the umbilicus, a distinct notch was palpable. The tumour was dull on percussion, though there was some resonance in the loin behind the tumour. The liver was not enlarged, and there was no enlargement of any lymphatic glands. The heart and lungs were normal. A diagnosis was made of simple hypertrophy of the spleen. Operation was not considered necessary unless the tumour gave trouble, and iron, arsenic, etc., were prescribed.

The patient did not come again until September, 1909, when she stated that she had had occasional pain in the left side of the abdomen since 1902, but that during the past two months it had been very much worse, and the swelling had been getting larger. The pain down the left side of abdomen now occurred almost daily, and she was prevented from getting about with comfort. There had been two slight attacks of jaundice since her last visit. The general condition of the patient was much the same as before, but the tumour had slightly increased in size. A blood count showed 7,200 white cells, of which 85.5 per cent. were polymorphonuclears and 14.5 per cent. were lymphocytes.

On September 24th, 1909, the spleen was removed through an incision in the left semilunar line. The pedicle was ligatured in three portions with silk, and another ligature was tied round the whole pedicle after removal of the spleen. The abdomen was closed in layers without drainage. After operation the pulse rose to 128 per minute, and continuous saline injections were given per rectum. The temperature was raised to 100°—101° for about a fortnight, and there was slight jaundice for about a month. The patient went home about five weeks after operation, and has continued well since.

An examination of the blood three days after operation showed :—

| | |
|---|---|
| Red blood cells .. .. .. .. .. .. | 2,160,000 |
| White blood cells .. .. .. .. | 23,800 |
| Polymorphonuclears .. .. .. .. | 82 per cent. |
| Transitionals .. .. .. .. .. | 3 per cent. |
| Large lymphocytes .. .. .. .. | 3 per cent. |
| Small lymphocytes .. .. .. .. | 11 per cent. |
| Eosinophils .. .. .. .. .. .. | 1 per cent. |

Before the patient left the red cells had risen to 5,000,000, and the white cells had dropped to 16,250.

The tumour weighed 1 lb. 13 oz. after some ounces of blood had drained away from it. Dr. Walker Hall reported on its structure as follows :—" There is general hypertrophy of all the tissues. No sign of lymphadenomatous change, no evidence of any growth. Capsule and trabeculæ thickened. Follicles swollen and active. Splenic pulp rather more hemorrhagic than usual. One or two small foci of calcareous deposit. The cells seem of the usual varieties."

The irregular temperature which occurred after operation in this case has been noted by other observers, and the diminution in the number of red blood cells and increase in the number of white cells referred to above seems to be a fairly common occurrence which lasts, on an average, about two months.

The intermittent attacks of jaundice from which the patient suffered has also been found in other cases, especially when splenoptosis has existed.

Considering the spleen is normally a somewhat fixed organ, it is surprising what an amount of mobility it may acquire. It is quite common for a wandering spleen to be found in the true pelvis, and its ability to travel into different parts of the abdomen has often been the cause of diagnostic errors.

For the correct appreciation of splenic disorders it is therefore necessary to exclude other abdominal organs, particularly the ovaries and left kidney, aided by the recognition of the characteristic notch and shape of the spleen.

Kelly truly says that no operator should remove a spleen without a previous general and searching examination, while a knowledge of the blood-findings is especially imperative.

